# Migration and horizontal gene transfer divide microbial genomes into multiple niches

**DOI:** 10.1038/ncomms9924

**Published:** 2015-11-23

**Authors:** Rene Niehus, Sara Mitri, Alexander G. Fletcher, Kevin R. Foster

**Affiliations:** 1 Department of Zoology, University of Oxford, South Parks Road Oxford OX1 3PS, UK; 2 Oxford Centre for Integrative Systems Biology, University of Oxford, South Parks Road, Oxford OX1 3QU, UK; 3 Department of Fundamental Microbiology, University of Lausanne, Lausanne CH-1015, Switzerland; 4 Wolfson Centre for Mathematical Biology, Mathematical Institute, University of Oxford, Radcliffe Observatory Quarter, Woodstock Road, Oxford OX2 6GG, UK; 5 School of Mathematics and Statistics, University of Sheffield, Hicks Building, Hounsfield Road, S3 7RH, UK

## Abstract

Horizontal gene transfer is central to microbial evolution, because it enables genetic regions to spread horizontally through diverse communities. However, how gene transfer exerts such a strong effect is not understood. Here we develop an eco-evolutionary model and show how genetic transfer, even when rare, can transform the evolution and ecology of microbes. We recapitulate existing models, which suggest that asexual reproduction will overpower horizontal transfer and greatly limit its effects. We then show that allowing immigration completely changes these predictions. With migration, the rates and impacts of horizontal transfer are greatly increased, and transfer is most frequent for loci under positive natural selection. Our analysis explains how ecologically important loci can sweep through competing strains and species. In this way, microbial genomes can evolve to become ecologically diverse where different genomic regions encode for partially overlapping, but distinct, ecologies. Under these conditions ecological species do not exist, because genes, not species, inhabit niches.

## Abstract (web summary)

Horizontal gene transfer is central to microbial evolution. Here, the authors develop an eco-evolutionary model and show that migration can greatly promote horizontal gene transfer, which explains how ecologically-important loci can sweep through the species in a microbial community.

## Graphical abstract

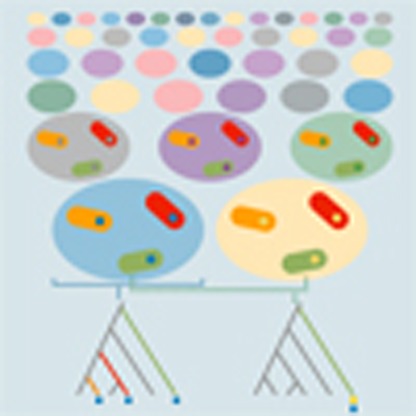

Microbes survive and reproduce in an extremely wide range of environments, from hydrothermal vents^1^ through marine snow^2^ and soil^3^, to host associations such as the human microbiome^4^. Within and between such environments, microbial genomes differ widely both in terms of allelic diversity and gene content^5^,^6^,^7^. At the heart of this genetic diversity is the ability of microbes to gain both homologous and non-homologous DNA via horizontal gene transfer. These transfers appear to occur in almost all prokaryotic lineages and have significant impacts on both bacterial and archaeal genomes^8^.

Horizontal transfer is considered central to the ability of cells to adapt to new ecological conditions including clinical or environmental settings that contain antibiotics^9^,^10^. Recent empirical work suggests that these transfers can spread a single beneficial allele horizontally through a microbial community—where the allele can either represent a single gene or a small group of genes—with the result that an otherwise diverse microbial community becomes genetically identical in a certain genomic region^9^,^11^,^12^,^13^,^14^,^15^. However, the rates at which gene transfers occur are thought to be extremely low, with asexual reproduction a much faster process. Competition between strains and species within a patch, therefore, will mean that a beneficial allele can spread much more quickly via whole-genome vertical transmission than horizontal transmission, which should prevent horizontal sweeps^16^,^17^,^18^,^19^. One way that genes can transfer horizontally is if they hop between ecologically distinct populations that do not compete^20^,^21^,^22^. However, the prevalence of competition within microbial communities^23^,^24^ suggests that vertical sweeps should remain a barrier to horizontal sweeps. And yet, the experimental evidence for horizontal sweeps comes from communities of phylogenetically related strains^9^,^11^,^12^,^13^,^25^ where ecological competition is likely to be significant.

A major question then is how horizontal transfer can so strongly impact microbial communities and cause the observed horizontal sweeps. Answering this question is necessary to understand microbes and how they evolve, both in nature and in the clinic. In a recent study, Takeuchi *et al*.^26^ provide an explanation for horizontal sweeps via negative frequency-dependent selection acting on other loci in the genome. Frequency-dependent selection can prevent full genome-wide selective sweeps and allow time for genetic transfer. Here we show that horizontal sweeps can occur without the need for negative frequency-dependent selection. We develop a series of models of a microbial community in which all cells compete for the same resource. We use these models to study both the rate of horizontal gene transfer and the impact that this transfer has on the genomics of the focal community. Our work reveals a missing ingredient that can explain horizontal sweeps: migration. When including immigration in our models, we find that the highest rates of horizontal transfer will occur for ecologically important traits that are under positive natural selection. The result is a genome where selected regions become partially decoupled from the ecology of the remaining loci such that different parts of the genome map to different niches.

## Results

### Overview

We are interested in understanding how a beneficial trait can spread horizontally through a microbial community. We build on a previously modelled scenario^18^,^27^ that considers a community of diverse strains that compete, and that will have the opportunity for genetic transfer^28^,^29^. Although we use the word ‘community' throughout, our model can also capture a set of strains from a single species, commonly referred to as a ‘population'. We follow the fate of a novel beneficial allele that is able to transfer horizontally between the genotypes in our community. Existing theory suggests that very little gene transfer should occur, because once a beneficial trait is picked up horizontally the carrier will rapidly outcompete the other strains in the patch before it has a chance to transfer the trait to other genomes^16^,^17^,^18^,^27^. However, previous models neglect the possibility that fixation of a trait can be delayed or prevented by migration of new cells into the community. We therefore begin by recapitulating previous predictions that competition suppresses gene transfer and we then show how making a single change, allowing immigration, can explain how horizontal genetic sweeps occur.

### Without migration adaptation by horizontal transfer is rare

We consider a community that lives in a focal patch, which can either be literally a single isolated patch or a set of similar patches that are themselves well connected by migration. At first we consider that this patch is largely isolated from the external environment, such that there is negligible migration between the community of the patch and the world at large. This scenario could correspond, for example, to communities living in hosts where there is limited superinfection with new strains and species over time^30^. We focus on those strains in the patch that are ecologically interchangeable in the sense that they compete for a common limiting resource. Under these conditions, horizontal transfer has been predicted to be almost powerless compared with vertical selective sweeps^16^,^17^,^18^,^27^.

Our model follows the fate of a rare beneficial allele that first appears in a small subset of the community. Although non-carriers have fitness *w*=1, carriers of the adaptive allele have increased fitness *w*=1+*s*, where *s* is the local benefit of the trait (*b*) minus the cost of carrying it (*ɛ*). We assume that the benefit of the trait in the focal patch is always larger than its cost, so that *s* > 0. Any cell in the community lacking the gene has a chance of picking it up horizontally and it is the overall rate of this process in the community that we are interested in. Not all loci can transfer their phenotypes horizontally^31^,^32^,^33^ and we focus here on those loci where horizontal transfer is possible. In addition, genetic transfer is typically relevant only for phenotypes that lie outside the range of physiological responses or short-term evolution by *de-novo* mutation^34^,^35^. Examples of such phenotypes—transferable and otherwise hard to achieve—include toxin resistance genes^36^, virulence factors^37^ and heat shock proteins^38^.

**Figure 1:**
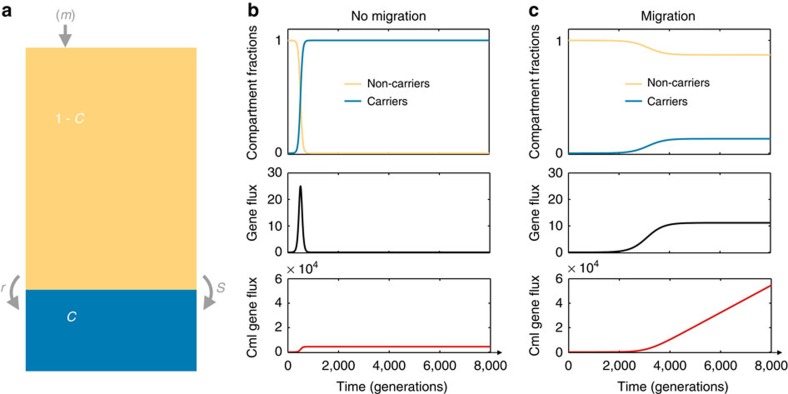
Migration greatly increases the potential for horizontal genetic transfer of a beneficial allele. (**a**) The compartment model. Sub-communities *C* (blue) and 1−*C* (yellow) represent the fractions of allele carriers and non-carriers in the community, respectively. In the absence of migration, positive selection (*s*) causes a replacement of non-carriers by carriers and gene transfer (*r*) converts non-carriers into carriers. In the presence of migration, non-carriers continuously arrive at the patch in addition to the processes of positive selection and gene transfer. (**b**) No migration: the beneficial allele is rapidly fixed so that the horizontal gene flux becomes zero and the total amount of horizontal transfers since the beginning of the sweep, the cumulative gene flux (cml gene flux), stays constant. (**c**) With migration: the immigration supplies the system with non-carriers, resulting in ongoing gene transfer at a constant rate. Other parameters are *N*=10^8^, *s*=0.025, *r*=10^−6^, *m*=0 (**b**) and *m*=0.02 (**c**).

We want then to capture the horizontal spread of an allele in a community of microbes. Although there is relatively little theoretical work on genetic transfer and its impact, there is a long theoretical tradition of modelling the horizontal spread of infectious diseases, along with associated empirical tests^39^,^40^. These models, often known as compartment models, have proved to be a powerful way to capture the key processes underlying the horizontal spread of a focal trait. We therefore begin here with a simple compartment model (Fig. 1a) that allows us to identify the conditions that maximize horizontal genetic transfer, before extending and developing our predictions using other modelling approaches.

We study a focal community of constant size *N* that contains two sub-communities, adaptive gene ‘carriers' and ‘non-carriers'. Applying a deterministic continuum approach, the relative community size in each sub-community and the flux between them can be described by the ordinary differential equation (ODE)


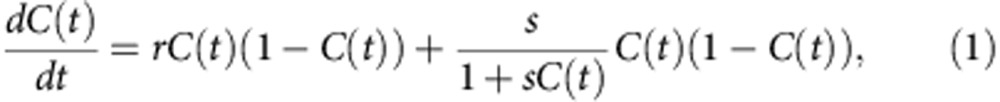


where *C*(*t*) is the fraction of carriers in the community at time *t*, 1−*C*(*t*) is the fraction of non-carriers and *r* is the rate of gene transfer from carriers to non-carriers (Fig. 1a). The growth of compartment *C* due to selection is given through the term *s*/(1+*sC*(*t*)). The term *rC*(*t*)(1−*C*(*t*)) captures the transition of cells from being a non-carrier to carrier through horizontal gene transfer. The proportion of trait carriers has two steady states (C*) when *s* or *r* are non-zero, given by


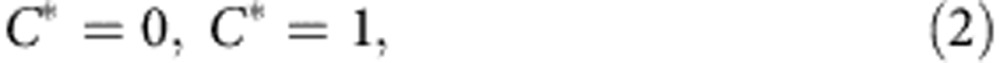


with C*=0 being an unstable and C*=1 being a stable equilibrium (see Supplementary Methods). Therefore, for any non-zero initial number of carriers (*C*(0) > 0) the fraction *C*(*t*) will increase until fixation of the focal beneficial allele (C*=1) at steady state. A key model parameter for our further analysis is the probability that a carrier cell transfers the focal allele to a non-carrier in any generation, *r*. Gene transfer rates in natural communities remain largely unknown. Previous theoretical work used a relatively low and conservative estimate for transfer rate of 10^−6^ per gene per generation^33^, which corresponds to our transition rate (*rC*(*t*)(1−*C*(*t*))). However, recent studies suggest that the rates of horizontal transfer can be much higher than such estimates^41^,^42^. We therefore consider a range of rates, 10^−6^ ≤ *r* ≤ 10^−4^, which goes either side of 10^−6^ per gene per generation (using *r*=10^−6^ guarantees that the transition rate *rC*(*t*)(1−*C*(*t*)) is <10^−6^). Genetic transfer can occur both by homologous and non-homologous recombination. Although we phrase our results here in terms of the former, our conclusions should apply to both mechanisms.

What then defines the rate of horizontal gene transfer in the whole patch? We can calculate this rate by multiplying the transition rate from carriers to non-carriers, *rC*(*t*)(1−*C*(*t*)), by the community size *N* to obtain the community-level rate of gene transfer, which we term horizontal gene flux (*rC*(*t*)(1−*C*(*t*))*N*). This horizontal gene flux spreads the beneficial gene without removing cells, whereas the vertical gene flux, given through *sC*(*t*)(1−*C*(*t*)), spreads the gene by removing non-carrier cells.

**Figure 2:**
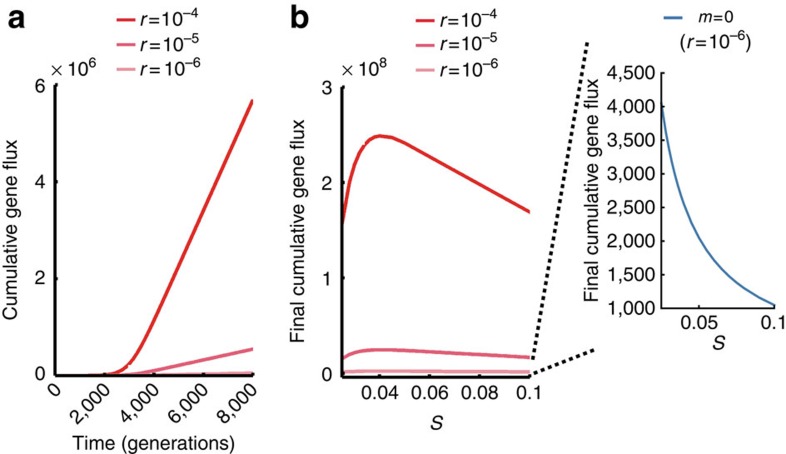
Migration allows horizontal transfer even with significant positive natural selection for a transferred trait. (**a**) Gene transfer events accumulate over time when immigration is possible, with increased gene transfer rates increasing the horizontal gene flux. (Selection pressure *s*=0.025). (**b**) Cumulative horizontal gene flux measured at *t*=10^5^ across different selection pressures (left-hand side plot) and for different rates of migration (right-hand side inset). With migration (left-hand plot, *m*=0.02), the cumulative gene flux peaks for intermediate selection pressure (*s*=0.04 with ∼2.5 × 10^6^ transfer events) and remains significant even for stronger positive selection (*s*=0.1 with ∼1.7 × 10^6^ transfer events). Without migration (inset plot, *r*=10^−6^), gene transfer remains extremely infrequent and peaks at the lowest selection pressure with only around 4,000 transfer events. In all plots, we calculate cumulative gene flux for *N*=10^8^ and *C*(0)=1,000/*N*.

As the selective sweep for the focal trait proceeds, the horizontal gene flux initially increases up to a maximum at a 1:1 ratio of donors and recipients (*C*(*t*)=1−*C*(*t*)=0.5) before decreasing back down to zero at allele fixation (Fig. 1b). The overall impact of this process can be quantified from the integral of the gene flux, which gives the expected number of horizontal gene transfer events over time (cumulative gene flux, see Methods). How is this cumulative gene transfer affected by the strength of positive selection? Plotting cumulative gene transfer against selection pressure shows that strong selection regimes minimize the effects of horizontal transfer, because the time window during which this transfer can occur is short (Fig. 2b, inset). Our model then recapitulates previous conclusions that, given a small rate of genetic transfer, only weakly selected traits can undergo significant horizontal transfer by slowly sweeping through a community^18^. However, weakly selected traits are, by definition, relatively unimportant for the ecology and evolution of their carriers. In contrast, many successful horizontally transferred traits, such as antibiotic resistance genes, appear to be both functionally important and under significant positive natural selection^29^,^43^,^44^ and these are the traits that we are interested in here.

### With migration adaptive traits transfer horizontally

Previous models of genetic transfer have not considered a key feature of microbial life, migration, which has the potential for important effects on population dynamics^45^,^46^. We next introduce migration into our model and we study its effect on our predictions. We now assume that our focal patch is separated, but not completely isolated, from its external environment so that there will be a limited but ongoing exchange of cells. For example, this focal patch could be a nutrient particle in ocean water, a mammalian host or tree hole. As discussed for our no-migration model, the ‘patch' can also represent a set of connected patches that all select for the same horizontally transferred trait, that is, a set of particles, hosts or tree holes. The key is that we now allow there to be immigration from other regions that do not select for the focal trait. Accordingly, we assume that all immigrating cells that arrive from outside the focal patch (or patches) lack the trait (but we relax this assumption below). For example, the focal allele might provide resistance to a toxin that is specific to the focal patch. Some immigrating cells will be unable to establish themselves in the focal community due to a mismatch with general ecological characters, such as nutrient conditions, temperature and alike. Other migrants might not be compatible with the selected locus and are unable to adapt. However, both of these effects will lead to fast extinction of these migrants and we can account for both by varying the migration rate, where an increase in the frequency of non-viable strains corresponds to a reduced migration rate. Extending our model to include migration gives


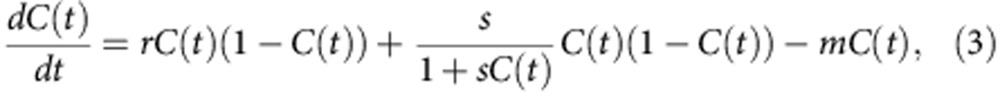


where *m* is the migration rate, given as the fraction of cells that is replaced through migrators landing and replacing them per unit time, and *mC*(*t*) is the replacement of trait carriers only (Fig. 1a). If the basic rate of genetic transfer is very small (*r* ≈ 0), the steady-state proportions of cells carrying the focal trait (C*) are given approximately by


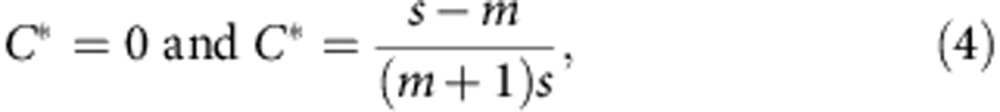


with C*=0 defining an unstable equilibrium and C*=(*s*−*m*)/((*m*+*1*)*s*) a stable equilibrium (see Supplementary Methods). Therefore, given an initial non-zero number of carriers, the system will reach the second equilibrium (C*=(*s*−*m*)/((*m*+*1*)*s*)). A key implication of this expression is that with non-zero migration (*m* ≠ 0) the selected trait will now reach a steady state before it has been fixed in the community (C* < 1), because migration continuously brings new genotypes into the system. These migrators mean that opportunities for genetic transfer remain after the initial selective sweep has occurred. Migration stops the selective sweep before it can complete as a classic selective sweep^47^ and instead there is a second longer-lasting incomplete sweep. Indeed, horizontal transfer now occurs as long as the community persists, which greatly increases its potential effects (Figs 1c and 2a). Migration rates are commonly considered to be high in natural microbial communities, as cells can be so easily dispersed, but exact rates are difficult to assess. In these first models, we use a relatively high rate of *m*=0.02, which corresponds to 2% of cells being replaced by incoming cells in each generation. However, we show in the next section that our conclusions are robust for a range of possible migration rates, just so long as migration does not overpower natural selection (*m*<*s*).

With migration in the model, the relationship between the strength of natural selection and the cumulative gene transfer is fundamentally changed. Now, horizontal transfer peaks for traits under intermediate selection pressure (Fig. 2), whereas without migration it peaks at minimum selection strength (Fig. 2b, inset). This means that migration greatly increases the potential for horizontal sweeps of ecologically important traits that are associated with significant positive selection pressures, for example, a trait that provides a fitness advantage of >10% (Fig. 2). The sweep occurs in spite of the fact that vertical flux remains the dominant mode of transmission in the community; even modest rates of natural selection (*s*>10^−3^) are much greater than the expected rates of gene transfer (10^−6^<*r*<10^−4^).

We have assumed so far that incoming migrators lack the adaptive trait. However, there are clearly cases where new cells may be pre-adapted and carry the focal trait. When will this occur and how does it change our predictions about horizontal gene flux? To investigate this, we consider an extended model that explicitly captures the external environment as an additional compartment where the focal trait is disfavoured by natural selection (Supplementary Fig. 1). Cells from the focal patch can leave and enter the surrounding environment and, equally, cells can return from the surrounding environment into the focal patch. As before, our ‘focal patch' can also represent a set of connected patches that all select for the same focal trait, which are surrounded by the wider external environment that does not favour the trait.

Our extended model makes the same predictions as our original model with migration whenever the external environment is large relative to the focal patch (Supplementary Figs 2 and 3). This is intuitive: a large external environment means that the focal gene is likely to be lost outside the focal patch before a cell returns, such that few or no immigrants will possess the focal trait. By contrast, when the external environment is itself a small patch, the focal patch and the external environment converge to act as a single patch in which the focal gene reaches fixation with limited horizontal gene transfer (as seen in the no-migration model above, Supplementary Figs 2 and 3). Another way to view the size of the external environment is as a proxy for the rarity of the focal niche: a large external environment that selects against the focal allele means that the focal niche is relatively rare. For the rest of the study, we focus on this case where a focal niche is rare relative to the environment from which immigrants arrive, such as a niche that selects for resistance to a specific antibiotic^48^. Under these conditions, the great majority of immigrants will be non-adapted.

### Horizontal transfer divides the genome into distinct niches

We have shown that migration from outside of a focal patch greatly increases the potential for gene transfer in microbial communities. However, is this increased transfer important for the ecology and evolution of microbes? Specifically, we are interested in whether the rate of horizontal transfer is sufficient to generate a horizontal selective sweep whereby a particular allele moves horizontally through a diverse community of microbes. To address this question, we next investigate how gene transfer with migration affects genomic diversity, at both the horizontally transferred and the non-transferred regions of the microbial genomes. Although compartment models allow us to follow the dynamics of horizontal transfer and identify the population processes driving a horizontal sweep, these models are not well suited to follow genomic effects. We therefore next develop a coalescence model to capture the genetic effects of genetic transfer, selection and migration probabilistically.

**Figure 3:**
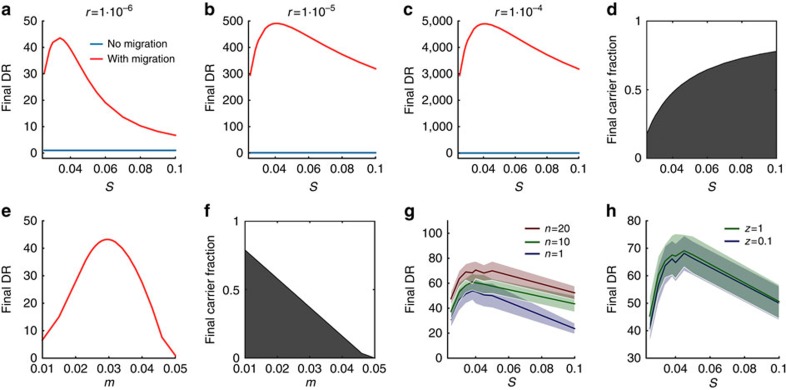
Migration enables horizontal genetic sweeps. We plot the diversity ratio (DR) as a function of the strength of natural selection (*s*) for *r*=10^−6^ (**a**), *r*=10^−5^ (**b**) and *r*=10^−4^ (**c**) as calculated from the coalescence model both with and without migration. With migration, the DR, which gives the strength of the selective sweep, peaks at intermediate selection pressure (*s* ≈ 0.04), whereas (**d**) the fraction of cells carrying the same adaptive gene increases monotonically with selection pressure, where for the different transfer rates the curve of the final carrier fraction is approximately identical, reaching about 80% for all three transfer rates. Parameters are *t*=5 × 10^6^, *N*=10^8^ and *C*(0)=1,000/*N*, and either *m*=0 or *m*=0.02. (**e**) The effect of migration rate (*m*) on the diversity ratio (DR) and (**f**) the effect of migration rate on the fraction of the community that carry the trait, for all three transfer rates (*s*=0.05, *t*=5 × 10^6^, *N*=10^8^ and *C*(0)=1,000/*N*). (**g**) The individual-based model shows the effect of ecologically different migrants for an increasing number of ecological niches (*n*=1, 10 and 20, and *z*=0.2). (**h**) We also plot (for *n*=20) the DR against selection pressure for two different levels of ecological specialization: cells competing twice as much in their own niche than in other niches (blue line) and cells that compete entirely in their own niche (green line). Other parameters are *N*=10^6^, *t*_end_=10^5^, *r*=10^−4^, *C*(0)=1,000/*N.* Results are averaged over 50 simulations and the s.d. is given by the transparent areas.

Our new model assesses the impact of genetic transfer in terms of how much it can decouple evolution at the genetic locus of the beneficial allele (focal locus) from the rest of the genome (background genome). We determine this effect by comparing the diversity at the focal locus (*D*_f_) to the diversity at the background genome (*D*_bg_), in the diversity ratio DR=*D*_bg_/*D*_f_ (for details, see Methods). Without migration, the DR changes little over time and remains close to one (Fig. 3a–c). Consistent with the predictions of our first model, we see very little effect of horizontal transfer when there is no migration. Genetic transfer is largely powerless to evolve the focal locus independently of the background genome and the two remain locked together in a vertical selective sweep that purges diversity in both genetic regions.

We next consider the case where there is immigration into the focal patch. Now, the behaviour of the model is very different. We find a wide range of parameter values for which the combination of positive natural selection, migration and horizontal transfer largely purge diversity at the focal locus, while leaving significant variability in the background genomes (high DR, Fig. 3a–c,e). The result is that the majority of cells carry the same adaptive allele (high fraction of carriers, Fig. 3d,f), while their background genomes remain diverse. The relative proportion of adaptive gene carriers increases monotonically with a stronger selection pressure (Fig. 3d) and with a decreasing migration rate (Fig. 3f). The DR is maximal when migration rate and positive selection are in a balanced regime. That is, some immigration is needed for an effective horizontal sweep to occur but, as seen in results from classical population genetics^49^, if migration is too strong (*m*>*s*) then it will overpower selection and prevent adaptive evolution (Fig. 3e,f). As a result, the horizontal gene sweep is most effective at intermediate positive selection pressures (Fig. 3a–c).

This result has major implications for the evolution of microbial communities in the face of horizontal transfer. Positive natural selection is no longer a barrier to the horizontal spread of a trait. Instead, the impacts of genetic transfer are greatest for ecologically important traits that are under positive natural selection. In this way, a horizontally transferred trait with its own specific ecology is able to move through a diverse set of strain backgrounds. As we discuss below, a key implication of this uncoupling is that genomes can become ecologically diverse in the sense that the ecology of the focal locus and the rest of the genome are overlapping but distinct.

### Ecological division of the patch promotes horizontal sweeps

We have so far focussed on an ecologically cohesive community, because the potential for diverse genotypes to compete ecologically is clear^23^,^24^,^50^ and also because previous work suggests that competitive exclusion in a community is the worst-case scenario for gene-specific horizontal sweeps^16^,^17^,^18^,^27^. For this reason the above results should be conservative in their estimates of how migration promotes horizontal sweeps. However, we can use an individual-based model to relax competition within the community and study the consequences for gene sweeps and genomic diversity.

We introduce ecological differences between genotypes by introducing *n* different background niches in our focal patch, which could represent specialization on different resources. Each incoming cell then belongs to one background niche but also partly competes within the other niches. We denote the extent to which a cell competes within its assigned niche as *z*, whose value lies between 0 and 1, where *z*=1 corresponds to a cell competing purely in its own niche and *z*=1/*n* corresponds to a cell competing equally in all niches (see Methods). Our simulations show that the genetic effect of horizontal gene transfer increases with an increasing number of distinct niches (*n*) in the community (Fig. 3g) and gene transfer is also increased by a stronger separation of the cells into separate niches (increasing *z*, Fig. 3h). This result is intuitive: the ecological subdivision reduces competition between cells in the different niches and thus reduces the loss of diversity in the background genome during the selective sweep. This effect of ecological subdivision agrees with the study of Majewski and Cohan^20^ who found that gene transfer has greater impact if communities were subdivided into a number of completely non-competing lineages (or ‘ecotypes'^21^). Another way to view the effect of ‘niches' in our model is in terms of negative frequency-dependent selection that prevents any one genotype from completely dominating the focal community^14^, which was the subject of a recent study by Takeuchi *et al*.^26^

In summary, our results suggest that an influx of diverse and non-adapted migrator genotypes can greatly increase the effect of genetic transfer on microbial communities. It does so in at least two ways. First, migration adds new gene recipients that, even after the beneficial trait is established, enable continued gene flux. Second, migration may introduce new genomes that increase ecological subdivision (*n* > 1). Both of these processes constrain the impact of vertical selective sweeps, but within ecologically cohesive communities (low *n*) it is the addition of new gene recipients that is critical for horizontal sweeps. The result is that most species and strains can evolve to be identical at the locus under positive selection, while the rest of the genomes are highly diverse.

## Discussion

**Figure 4:**
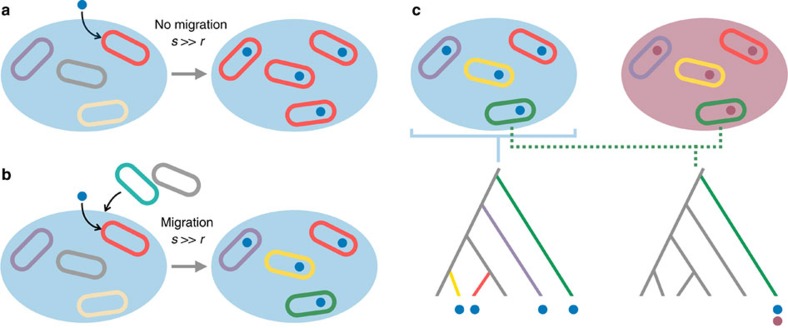
Horizontal transfer creates multiple ecologies within one microbial genome. (**a**) The ecotype model^70^. A selected trait (blue dot) causes a selective sweep of the genome in which it first appears (red cell), wiping out other genomes if selection strength (*s*) for the trait is much larger than its gene transfer rate (*r*). (**b**) Divided-genome model. Immigration of new genotypes causes a horizontal genetic sweep because incoming genotypes can pick up the selected trait. The trait is now found in diverse genomic backgrounds, which can have ecologies that are distinct from both the focal locus and from each other. (**c**) Genomic regions can display high or low diversity depending on the ecological basis of sampling. The two circles containing the cells represent patches with loci-specific sweeps. Sampling from a single patch that selects for a horizontally transferred locus (blue dots) will capture cells that are diverse in their background genome phylogeny but homogeneous at the transferred locus (horizontal gene sampling, left-hand phylogeny). In contrast, sampling from a single background genome (green cells) will capture cells that are diverse in their horizontally transferred loci (background genome sampling, right-hand phylogeny).

Our models explain how horizontal sweeps of small stretches of DNA can occur in ecologically cohesive communities of microbes. The strains and species that compete within such communities are ideal candidates for horizontal transfer, because they live in close proximity and they can induce lysis in one another releasing DNA for uptake^51^,^52^. However, previous work suggests that transfer of a beneficial gene within competing communities should be limited by selective sweeps that propagate the allele vertically to fixation before significant horizontal transfer can occur^16^,^17^,^18^,^27^(Fig. 4a). Here we have shown that this prediction does not hold when one includes the possibility of immigration. Migration is a significant process in microbial ecology^45^,^46^ and allowing migration in our model results in large amounts of horizontal transfer that has the power to transform the genomics of the community.

Our models then provide an evolutionary explanation for the increasing number of sequencing studies showing that otherwise diverse microbial communities possess regions of the genome that contain very little diversity^9^,^11^,^12^,^13^,^53^. Further evidence of the processes we describe comes from the recent observation that mobile genetic elements can be enriched in their own niches, largely independently of their bacterial host^25^,^28^,^29^. Horizontal transfer then has the potential to make diverse and competitive strains coherent in an ecologically important phenotype, including key traits such as resistances to toxins^9^. Although our models explain how horizontal sweeps can occur, they also predict that the timescale required for a sweep is likely to be on the order of months to years (for example, 10^4^ to 10^6^ generations for a 30-min generation time), based on current estimates of genetic transfer rates^33^,^41^,^42^. A key prediction then is that horizontal sweeps are relatively slow compared with the canonical vertical sweep often seen in the laboratory^54^,^55^. Nevertheless, the timescales of horizontal sweeps remain extremely short compared with phylogenetic timescales, and fit with data showing that genetically coherent microbial communities persist for years in the face of migration^56^ and vertical sweeps^57^. Recent work also suggests that the basic rates of gene transfer can sometimes be much higher than typically assumed^41^,^58^, which in our model will significantly reduce the timescales required for horizontal sweeps. This prediction contrasts with the horizontal gene sweep scenario described by Takeuchi *et al*.^26^, in which gene transfer rates need to remain low (<10^−6^), for single-gene sweeps to occur.

We have emphasized here how horizontal gene transfer can remove diversity at one locus relative to the rest of the genome in a microbial community. How is this result reconciled with the notion of horizontal transfer as a way to generate diversity, in particular in the form of the much-discussed accessory genome^13^,^14^,^53^,^59^? Our analyses explain how genetic transfer can be seen to generate diversity in some studies, while removing diversity in others. This effect can be illustrated by considering two contrasting examples. First, if an experimenter samples in a specific patch that, as in our model, selects strongly for a particular horizontally transferred trait, then the data may show evidence of the horizontal sweep that removed variability at the focal locus relative to the rest of the genome (horizontal gene sampling, Fig. 4c)^11^,^12^,^53^. In contrast, if an experimenter samples one species such as *Escherichia coli* across different locations, then its background genome is likely to cross many niches for different horizontally acquired loci. In this case, horizontal transfer will be a process that mostly generates diversity relative to the core genome (background genome sampling, Fig. 4c)^60^,^61^. Arguably then, what is considered the ‘accessory' region of a genome will depend on the ecological basis for sampling^15^.

Our work speaks to the fundamental question of how microbial genotypes map to ecology^14^,^62^,^63^. A key result from our model is that the highest rates of transfer occur for loci that are under significant positive natural selection: loci that are important for the ecology of a cell. Horizontal transfer, therefore, can enable a particular locus to accumulate in a local environment to which it is evolutionarily adapted, without the rest of the genome evolving in the same way. An interesting corollary is that a single cell carrying such loci will become ecologically diverse, in the sense that its genome can evolve to become a community of genetic regions with multiple partially overlapping, but distinct, ecologies. This idea of distinct ‘gene ecologies'^64^ has recently been discussed in light of the microbial species question^25^,^29^,^64^,^65^,^66^. Our model explains how distinct gene ecologies are possible, as well as identifying the conditions required for them to occur (Fig. 4c). We show that, with sufficient migration, an ecologically important trait can readily decouple itself from any one genetic background via horizontal transfer. When this occurs, a microbial niche is defined at the sub-genomic scale so that ecological species concepts will no longer map to the whole organism but rather to a subset of any one genome.

## Methods

### Continuous model

We model the dynamics of a selective sweep with opportunity for genetic transfer and migration using an ODE. This equation describes the community in our selective patch where there are two sub-communities: carriers and non-carriers of the selected trait. The community is assumed to have a constant number of cells (*N*) with varying fractions of beneficial gene carriers (*C*) and non-carriers (1−*C*). For simplicity, we assume the community to be well-mixed and we do not consider stochastic effects (we relax this later). The dynamics and steady-state levels of carriers in the community are described by equations (1, 2, 3) in the main text. To capture the gene flux through horizontal transfer in a given community, we define a composite parameter that is the rate of transfer of the selected trait in the whole community:


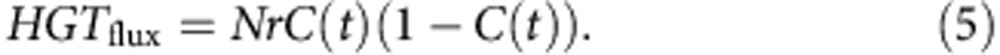


This horizontal gene flux is maximized at equal number of donors and recipients (*C*=0.5). At steady state of the system, the gene flux is given by:


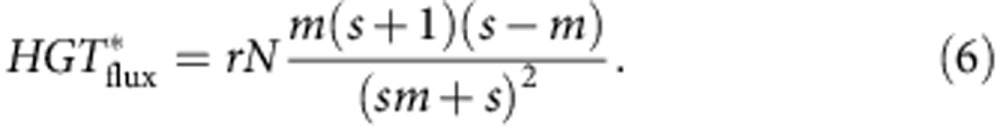


Figure 1b,c show plots of *C*, 1−*C* and the horizontal gene flux over time with and without migration. Gene transfer events accumulate over time and ultimately cause sweeps of single loci or small groups of loci. As the horizontal gene flux gives the rate of gene transfer events, the integral of this flux over a time interval gives the expected number of transfer events in this time. To obtain the numerical solutions of the cumulative gene flux plotted in Figs 1 and 2, we employ the rectangle rule in MATLAB. Table 1 provides a summary of the parameters present in this model and a more detailed description and analysis of the model are given in the Supplementary Methods.

### Coalescence model

We use a coalescence approach to model the genomes in our focal patch under influence of the selective sweep in combination with horizontal transfer and migration. We first simulate the fraction of selected gene carriers (*C*) in the time interval *t*∈[0, *t*_end_] using the ODE of our continuous model. With the simulated values of *C*(*t*) we then compute the coalescence process of two homologous loci, to determine the expected diversity in their genome site. For the diversity in the background genome, we consider two random background loci at time *t*=*t*_end_ and for the diversity in the focal locus we consider two focal loci at *t*=*t*_end_. We then go backward in time until *t*=0, while updating the probabilities of the two loci being in a given state. The loci can take the following states:

State 11: Both loci are in two distinct individuals that are both carriers.

State 00: Both loci are in two distinct individuals that are both non-carriers.

State 01: Both loci are in two distinct individuals where one is a carrier and the other one a non-carrier.

State 1: The two loci are coalesced in one individual that is a carrier.

State 0: The two loci are coalesced in one individual that is a non-carrier.

State *m*: At least one of the loci is in a migrating individual outside the patch.

We simulate the change of all six probabilities backward in time, until *t*=0, by solving a set of coupled ODEs given in the Supplementary Methods. We obtain the diversity in the focal locus *D*_f_ and the diversity of the background genome *D*_bg_. We measure the power of the horizontal gene sweep using the ratio of the diversity in the background genome over the diversity in the focal locus and we call this the DR given by:


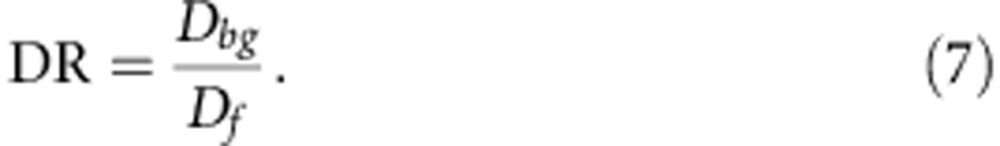


### Individual-based model

We develop an individual-based model of our selective patch to confirm the predictions of our coalescence model and to be able to change the ecological details of the patch. The simulated patch contains a fixed number of cells (*N*), where each individual cell is described by a set of three numbers representing the focal locus (transferrable), the genotype of the remaining background genome (non-transferrable) and the niche/resource association of the genotype. The background genotype can take any positive integer, which matches the focal locus for cells at the beginning of the simulation and for migrating cells. The focal locus can take the adapted state 1 or alternatively any other positive integer for non-adapted cells. We simulate the ecological competition of the cells in *n* different niches in the patch similar to the *symsim* model by Friedman *et al*.^67^. Each cell obtains resources from an assigned niche and from the remaining niches as well. Thus, for a given cell *i* there is a vector of length *n* giving the cell's ecological fit to each niche. A cell's fit to its assigned niche is denoted by *z* ∈ [0,1], where *z*=1 means that a cell only competes in its assigned niche and *z*=1/*n* means that a cell competes in all niches equally. We then define the competitive weight *ω* of a cell *i* in niche *j* is as the product of its fitness *f*_*i*_ (*f*=1+*s* for carriers and *f*=1 for non-carrier cells) times its association with niche *j*, so that *ω*_*ij*_=*f*_*i*_*σ*_*i*,1_. Each of the *n* niches holds a resource share of *N*/*n* in each generation, so that a cell obtains resources from niche *j* proportionally to its relative competitive weight in this niche according to:


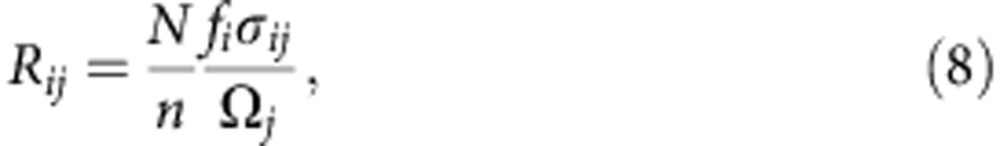


where *R*_*ij*_ is the amount or resources obtained by cell *i* from niche *j* and *Ω*_*j*_ is the summed competitive weight in niche *j* given by:


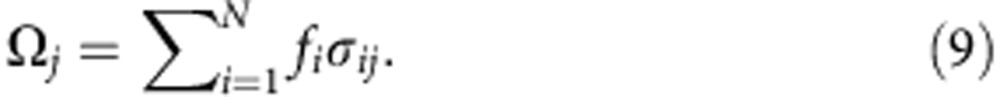


The total amount of resources obtained by cell *i* per generation is then given by:


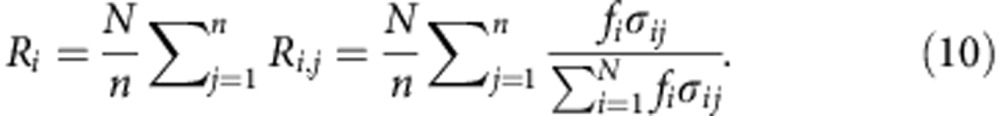


The resources of a cell determine the reproduction of a cell and we can use *R*_*i*_ as the mean number of offspring of a cell. We update the cell numbers stochastically using a Poisson distribution following a discrete time Wright–Fisher process^68^. Then, cells that lack the selected trait have a chance of acquiring the trait with a probability *C*(*t*)*r.* We implement the simulations using MATLAB and measure the horizontal gene flux and the diversities in different parts of the genome. The diversity is calculated as:


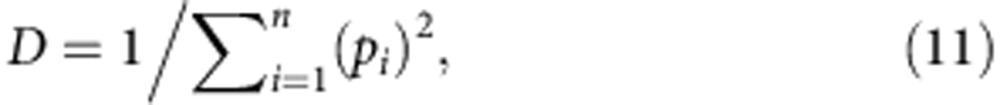


where *n* is the number of different locus variants present in the community and *p*_*i*_ is their respective proportion. This calculation is analogous to the effective number of species in a community (of order 2 (ref. 69)). We measure the genetic effect of the horizontal sweep as in the coalescence model using the DR given by:


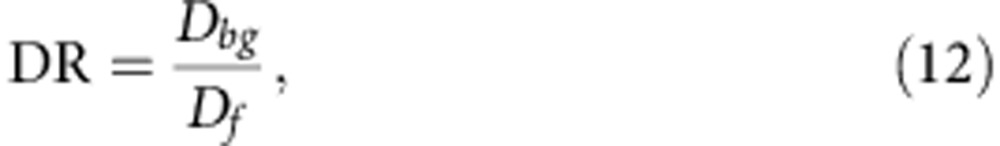


where *D*_bg_ is the diversity in the background genome and *D*_f_ is the diversity in the focal locus. More details of this simulation are given in the Supplementary Methods. We show that the results of our individual-based model and coalescence model match quantitatively, despite being fundamentally different models (Supplementary Fig. 4).

### Code availability

The MATLAB code of our individual-based model is available online via http://zoo-kfoster.zoo.ox.ac.uk.

## Additional information

**How to cite this article:** Niehus, R. *et al*. Migration and horizontal gene transfer divide microbial genomes into multiple niches. *Nat. Commun.* 6:8924 doi: 10.1038/ncomms9924 (2015).

## Supplementary Material

Supplementary Information Supplementary Figures 1-4, Supplementary Methods and Supplementary References

## Figures and Tables

**Table 1:** Parameters used in the compartment model.

Parameter	Range	Description
*N*	*Positive integers*	Carrying capacity of the community
*C*_N_	[0,*N*]	Total number of trait carriers in the focal patch
*C*	[0,1]	Fraction of carriers in the focal patch
*r*	[0,1]	Rate of gene transfer between trait carriers and non-carriers per generation
*s*	[0,∞]	Strength of positive selection, given as the fitness increase of allele carriers in the focal patch
*m*	[0,1]	Migration rate per generation time, given as a fraction of the patch community
