# Supplementary material for: Migration and horizontal gene transfer divide microbial genomes into multiple niches
Source: Nat Commun. 2015 Nov 23;6:8924. doi: 10.1038/ncomms9924 (PMC4673824; doi:10.1038/ncomms9924)
Supplement: Supplementary Information — Supplementary Figures 1-4, Supplementary Methods and Supplementary References [file ncomms9924-s1.pdf]

Supplementary Figures.

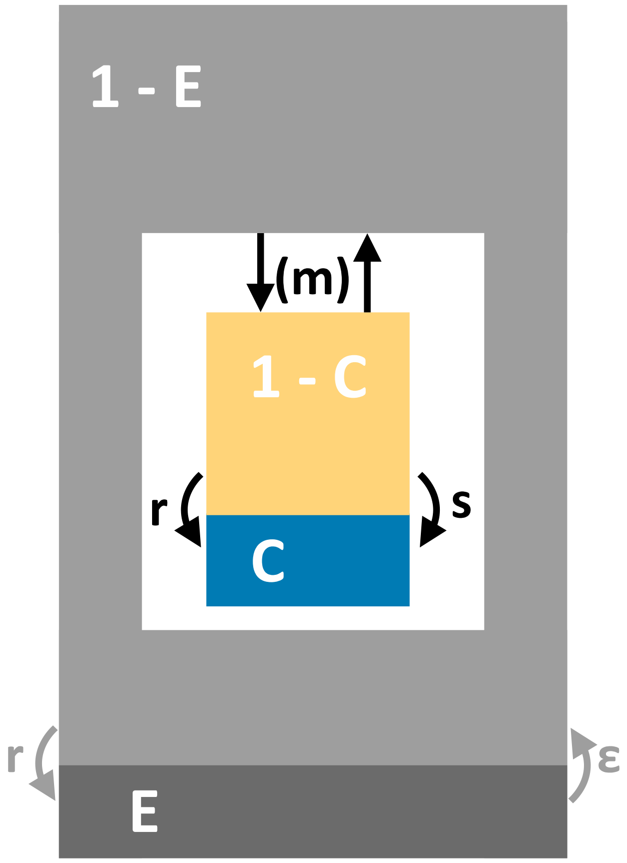

Supplementary Figure 1 | The extended compartment model. Sub-compartment *C* (blue) and 1-*C* (yellow) represent the fractions of allele carriers and non-carriers in the focal patch, respectively, and sub-compartment *E* (dark grey) and 1-*E* (light grey) represent the fractions of allele carriers and non-carriers in the surrounding environment, respectively. Gene transfer (*r*) converts non-carriers into carriers. Positive selection (*s*) causes a replacement of non-carriers by carriers in the focal patch, while the cost (ε) causes a replacement of carriers by non-carriers in the external environment. Migration (*m*) exchanges cells between focal patch and external patch.

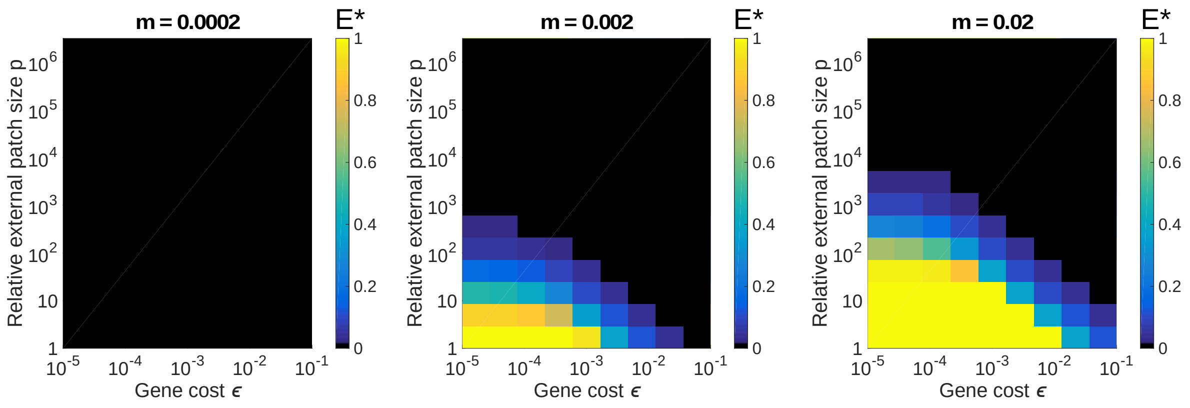

Supplementary Figure 2 | The focal trait is lost in large environments. Final fractions of gene carriers in the external environment calculated for different relative external patch sizes (*p*), gene costs (*ε*), and migration rates (*m*). The final fraction of gene carriers *E** is given as a colour from yellow (high *E**) to blue (small *E**) and with very small values ($E^* < 0.0001$) indicated in black. The plots show that the amount of external gene carriers becomes vanishingly small for $p > 10^4$. For lower values of *p* ($p < 10^4$) one observes that *E** becomes lower for higher gene costs *ε* and for lower migration rates *m*. The other parameters are, as in main-text Figure 1 and Figure 2, $N = 10^8$, and $C(0) = 1000/N$, $E(0)=0$, r = 10-6 and for each panel $s = 2m$.

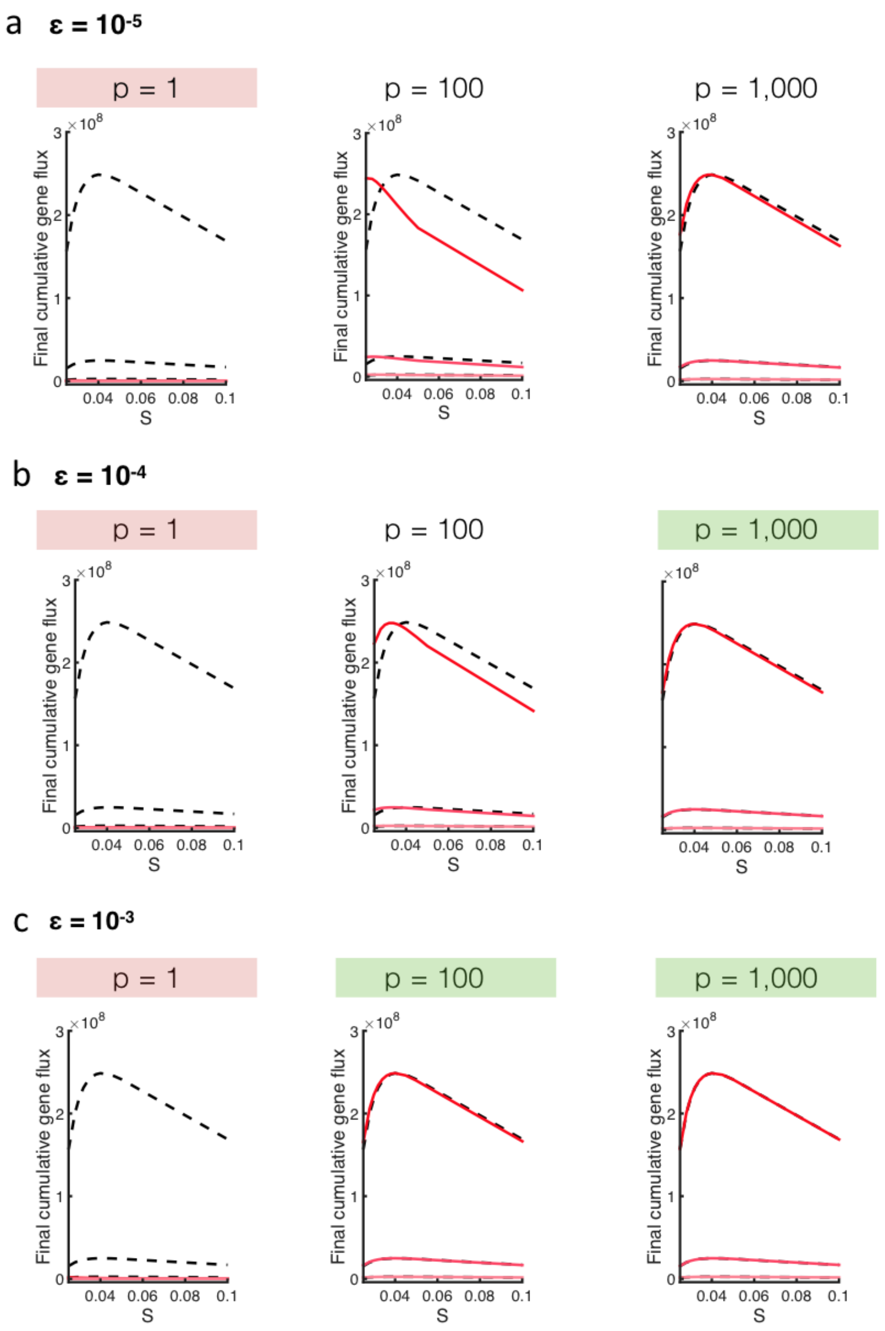

Supplementary Figure 3 | Comparison of the simplified model (Fig. 1a) and the extended compartment model (Supplementary Fig. 1). Graphs showing the final cumulative horizontal gene transfer over a range of positive selection pressures, as shown in Figure 2b. Here we show the results of the simplified compartment model (assuming $E(t) = 0$) in black and the results of the extended compartment model as red dotted lines for a range of p and ε. There are 3 different outcomes: for low

values of p and of ε, gene flux is very limited in the extended model (plots highlighted in red). For intermediate values of p and ε the gene flux of the extended model peaks at a different selection pressure than the simplified model, but with the same peak value (non-highlighted plots). For high values of p and of ε the gene flux of the two models are almost identical (gene flux of the extended model differs less than 1% from the simplified model, plots highlighted in green). The cumulative gene transfer is measured at $t = 10^5$ and the other parameters are $N = 10^8$, and $C(0) = 1000/N$.

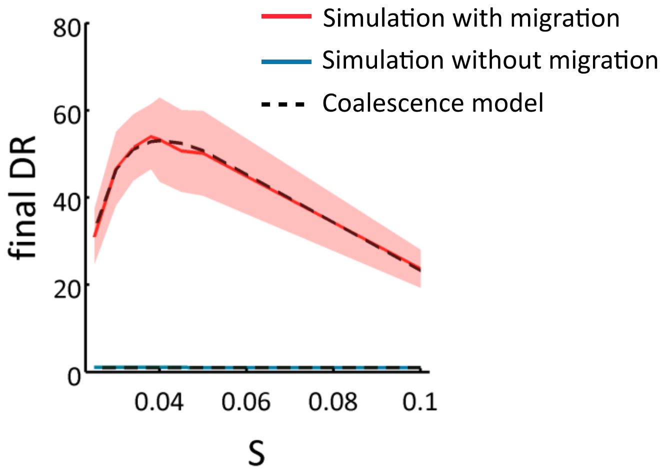

Supplementary Figure 4 | The Wright-Fisher simulations match the coalescence model. Graphs showing the final diversity ratio over a range of positive selection pressures calculated using the coalescence model (dotted black lines) and calculated using the individual-based model (red and blue lines). Simulation results are averaged over 50 simulations, and the standard deviation is given by the transparent area. The two different models show the same results. ($N = 10^6$, $t_{end} = 10^5$, $r = 10^{-4}$, $C(0) = 1000/N$, and either $m = 0$ or $m = 0.02$)

Supplementary Methods.

Here we present an extended version of the methods presented in the paper.

A) Continuous model

Here we introduce an extended version of the compartment model presented in the main text. This extended model captures how a focal gene spreads both inside a focal patch where it is under positive selection, and outside this patch. Outside the focal patch the cells do not benefit from the focal gene but pay a fitness cost when they carry it. For simplicity, we capture the external environment as a single patch that is connected to the focal patch by cell migration.

Our model is analogous to an SIR (Sensitive, Infected, Resistant) model, often known as a “compartment model”, which is commonly used to describe the dynamics of an epidemic by representing the flow between sub-communities ("compartments"). Our model contains two patches, the focal patch and the environment patch, each comprising two compartments: carriers ("infected") and non-carriers ("sensitive") of the selected trait. In the focal patch there are a constant number of cells (*N*) and varying fractions of beneficial gene carriers (*C*) and non-carriers (1-*C*), outside the patch there is a *p*-times larger number of cells (*pN*) and varying fractions of focal gene carriers (*E*) and non-carriers (1-*E*). For simplicity, each patch is well-mixed and we do not consider spatial effects. We also do not consider stochastic effects in this model but our later models do. The dynamics within both patches are determined by

the following three processes, which are considered to occur continuously in time, where one time unit is one cell generation:

1. Selection: A cell's fitness depends on whether it carries the focal gene or not and whether it lives in the focal patch or not. The general fitness term of a cell is $w = 1 - \varepsilon + \beta$, where ε is the cost that all trait-carriers experience and β is the benefit that only trait-carriers within the focal patch experience. We assume that the benefit of the focal trait in the selective patch is larger than its cost ($\beta > \varepsilon$) so that the net change in fitness is positive ($s = \beta - \varepsilon$, $s > 0$). This leads to the following fitnesses:

Carriers in focal patch: $w = 1 + s$, non-carriers in focal patch: $w = 1$

Carriers in environment patch: $w = 1 - \varepsilon$, non-carriers in environment patch: $w = 1$

2. Horizontal gene transfer: We let r be the probability that a carrier transmits the focal gene to a non-carrier per cell generation and we assume that horizontal transfer occurs according to the mass action principle: the transmission rate is proportional to both the fraction of recipients ($1-C$ or $1-E$) and the fraction of donors (C or E).

3. Migration: Migration works as an exchange of cells between focal patch and environment patch. The migration rate is specified through m, the fraction of the cells in the focal patch that is replaced randomly by individuals from the external patch landing in the focal patch per unit time. In the external patch cells from the focal patch replace a fraction of m/p per unit time.

The evolution of our compartment model can be described using two coupled ordinary differential equations (ODEs). We let $C_N(t)$ and $E_N(t)$ be the number of

carriers in the focal patch and the environmental patch, respectively, at time t. Due to the constant carrying capacities of the patches, the number of non-carriers is given through $N - C_N(t)$ for the focal patch and through $pN - E_N(t)$ for the environment patch. We can then describe the dynamics of the whole system by considering sub-communities C and E only. The rate of transitions from sub-compartment 1-C to C via horizontal gene transfer is given by $v_{HGT} = rC_N(t)\,(N - C_N(t))/N$. Similarly, in the environment patch the rate of gene transitions is given by $\omega_{HGT} = rE_N(t)\,(pN - E_N(t))/(pN)$. The rate of increase in size of carrier-compartment C due to positive selection is given by $v_{selection} = s/(1 + sC_N(t)/N)C_N(t)\,(N - C_N(t))/N$. In the environment patch the carrier-compartment E is reduced by selection at rate $\omega_{selection} = \varepsilon/(1 - \varepsilon E_N(t)/(pN))E_N(t)\,(pN - E_N(t))/(pN)$. Finally, the rate at which individuals in C are replaced through migration is given by $v_{migration} = mNC_N(t)(pN-E_N(t))/(pN) - mNE_N(t)/(pN)(N-C_N(t))/N=mN(C_N(t)/N-E_N(t)/(pN))$. In the external patch, migration increases the carrier-compartment E at rate $\omega_{migration} =mN(C_N(t)/N-E_N(t)/(pN))$.

In summary, the system evolves according to the system of ODEs

$$\frac{dC_N(t)}{dt} = v_{HGT} + v_{selection} - v_{migration},$$

$$\frac{dE_N(t)}{dt} = \omega_{HGT} - \omega_{selection} + \omega_{migration}, \quad \text{(A1)}$$

or

$$\frac{dC_N(t)}{dt} = rC_N(t)\frac{N-C_N(t)}{N} + \frac{s}{1+sC_N(t)/N}C_N(t)\frac{N-C_N(t)}{N} - mN\left(\frac{C_N(t)}{N} - \frac{E_N(t)}{pN}\right),$$

$$\frac{dE_N(t)}{dt} = rE_N(t)\frac{pN-E_N(t)}{pN} - \frac{\varepsilon}{1-\varepsilon E_N(t)/(pN)}E_N(t)\frac{pN-E_N(t)}{pN} + mN\left(\frac{C_N(t)}{N} - \frac{E_N(t)}{pN}\right). \quad \text{(A2)}$$

Dividing the first equation of equation system (A2) throughout by N and dividing the second equation throughout by pN yields, for the carrier-fractions $C(t) = C_N(t)/N$ and $E(t) = E_N(t)/(pN)$, the coupled equations:

$$\frac{dC(t)}{dt} = rC(t)\big(1 - C(t)\big) + \frac{s}{1+sC(t)}C(t)\big(1 - C(t)\big) - m(C(t) - E(t)),$$

$$\frac{dE(t)}{dt} = rE(t)\big(1 - E(t)\big) - \frac{\varepsilon}{1-\varepsilon E(t)}E(t)\big(1 - E(t)\big) + \frac{m}{p}(C(t) - E(t)). \quad \text{(A3)}$$

For a visualisation of our extended compartment model see Supplementary Figure S1. In the external patch, trait carriers are added through migration from the focal patch, and the trait spreads through horizontal transfer and is removed due to positive selection. Given a very small gene transfer rate, the key parameters that determine to what extend the trait is maintained in the external patch are the cost ε and p, the size of the external patch relative to size of the focal patch. Because microbial genome evolution by gene gain and loss is biased towards loss of unnecessary genes[1,2], we assume that ε is larger than the gene transfer rate r ($\varepsilon > r$). We explore in our model an according range of values for ε ($10^{-5} < \varepsilon < 10^{-1}$ in Supplementary Fig. 2 and $10^{-5} < \varepsilon < 10^{-3}$ in Supplementary Fig. 3). The migration parameter m determines the amount of cell exchange between the patches, and when m is very high it causes the two patches to behave close to a single, well-mixed patch. Because we assume that the focal patch and its environment are spatially separated, we consider $m = 0.02$ as a high migration rate and choose this to be the upper limit. We plot the fraction of carriers in the external patch at equilibrium (E^*)

for a range of parameters ε and p (see Supplementary Fig. 2). We determine E^* by solving equations (A3), in MATLAB until equilibrium is reached, with initial conditions $C(0) = 0.001$, $E(0) = 0$ and for different migration rates m and a selection pressure of $s = 2m$ so that the focal trait is fixed in the selective patch. Supplementary Figure S2 shows that when the external patch is much larger than the focal patch ($p > 10^4$), then the focal trait is extremely rare outside the focal patch ($E^* < 0.0001$). We begin by studying this case in detail, by setting $E(t) = 0$:

$$\frac{dC(t)}{dt} = rC(t)\big(1 - C(t)\big) + \frac{s}{1+sC(t)}C(t)\big(1 - C(t)\big) - mC(t), \quad \text{(A4)}$$

The steady states of this system for $r \approx 0$ is given approximately by:

$$C^* = 0 \ \ and \ C^* = \frac{s - m}{(m + 1)s}, \quad \text{(A5)}$$

with $C^*= 0$ defining an unstable equilibrium and $C^* = (s - m)/((m + 1)s)$ a stable equilibrium, given $s > 0$, according to linear stability analysis. Here and throughout, we use an asterisk to denote the steady-state value of a variable.

Table 1 provides a summary of the parameters present in this model.

Horizontal gene flux. To capture the gene flux through genetic transfer in our focal community, we define a composite parameter that is the rate of transfer of the selected trait in the whole community:

$$HGT_{flux} = NrC(t)(1 - C(t)). \quad \text{(A6)}$$

This horizontal gene flux spreads the beneficial gene without removing migrant cells, while the vertical gene flux, given through $sC(t)(1 - C(t))$, spreads the gene with removing migrants. The horizontal gene flux is maximized at equal numbers of donors and recipients ($C = 0.5$), and it thus depends on the patch conditions, including the initial number of trait carriers. At steady-state of the system, the horizontal gene flux then only depends on the selection pressure, the migration rate and the rate of gene transfer. In this case the horizontal gene flux is calculated by substituting equation (A5) for the steady-state fraction of non-carriers into equation (A6), giving

$$HGT^{*}_{flux} = rN\frac{m(s+1)(s-m)}{(sm+s)^2}. \quad \text{(A7)}$$

Analyzing this expression for the horizontal gene flux at steady-state, we find that there is no gene flux in the absence of migration ($HGT_{\text{flux}} = 0$ for $m = 0$). We also see that, for a given migration rate, the gene flux is highest at an intermediate selection pressure ($s = 2m/(1 - m)$).

Cumulative gene flux. Gene transfer events can accumulate over time and may ultimately cause sweeps of the focal locus. Because the horizontal gene flux gives the rate of gene transfer events, the integral of the flux over a time interval gives the expected number of transfer events in this time. To obtain the numerical solutions of the cumulative gene flux plotted in Fig. 1 and Fig. 2, we employ the midpoint rule.

Explicitly modelling immigration. We now move to explicitly modelling immigrating cells to relax the assumption that migrants never arrive pre-adapted in the focal patch and test how this changes our results. For this we return to equations (A3) and we use these equations to repeat the calculations of the final cumulative gene flux using different values for p and ε, and we compare the new results with our results from Figure 2b (Supplementary Fig. 3).

Very small fractions of external carriers (Supplementary Fig. 2, black areas) then mean that the system with external patch (Eq. A3) behaves like the simplified system (Eq. A4) and the latter is a good approximation of the former. In our next models, we explore the effects of the horizontal gene flux observed when migrants lack the adaptive trait ($E(t) = 0$) which corresponds to the case were the outside environment is much larger than the focal patch.

B) Coalescence model

We use a coalescence approach to model the genomes of our community under influence of a selective sweep in combination with genetic transfer and migration. We ask how these processes influence the evolution of genetic diversity in the focal locus and in the background genome. To answer this question we first simulate the fraction of selected gene carriers (C) in the time interval $t \in [0, t_{\text{end}}]$ using equation (A3). With the simulated values of $C(t)$ we can then compute the coalescence process for two homologous loci in order to determine the expected diversity in their genome site. For the diversity in the background genome, we consider two random background loci at time $t = t_{\text{end}}$ and for the diversity in the focal locus we

consider two focal loci at $t = t_{\text{end}}$. We then go backward in time until $t = 0$, while updating the probabilities of the loci being in a given state.

The loci can take the following states:

State *11*: Both loci are in two distinct individuals that are both carriers

State *00*: Both loci are in two distinct individuals that are both non-carriers

State *01*: Both loci are in two distinct individuals where one is a carrier and the other one a non-carrier

State *1*: The two loci are coalesced in one individual that is a carrier

State *0*: The two loci are coalesced in one individual that is a non-carrier

State m: At least one of the loci is in a migrating individual outside the patch.

We first explain how we apply the coalescence model to the background genome. When taking two random loci of the background genome at $t = t_{\text{end}}$, we know that these loci can be found in one of the first 3 states: *11* (with probability $P_{11}(t_{\text{end}}) = C(t_{\text{end}})^2$), *00* (with probability $P_{00}(t_{\text{end}}) = (1 - C(t_{\text{end}}))^2$), or *01* (with probability $P_{01}(t_{\text{end}}) = 2C(t_{\text{end}})(1-C(t_{\text{end}}))$). The probabilities for the remaining states are zero ($P_1(t_{\text{end}}) = 0$, $P_0(t_{\text{end}}) = 0$, $P_m(t_{\text{end}}) = 0$). We use these probabilities as our initial condition and simulate the change of all six probabilities backward in time, until $t = 0$, by solving the following set of coupled ODEs:

$$\frac{dP_{11}(t)}{dt} = -(p_{11\to 01} + p_{11\to 00} + p_{11\to 0} + p_{11\to 1})P_{11}(t),$$

$$\frac{dP_{00}(t)}{dt} = -(p_{00\to 0} + p_{00\to m})P_{00}(t) + p_{11\to 00}P_{11}(t) + p_{01\to 00}P_{01}(t),$$

$$\frac{dP_{01}(t)}{dt} = -(p_{01\to 00} + p_{01\to 0} + p_{01\to m})P_{01}(t) + p_{11\to 01}P_{11}(t),$$

$$\frac{dP_1(t)}{dt} = +p_{11\to 1}P_{11}(t),$$

$$\frac{dP_0(t)}{dt} = p_{11\to 0}P_{11}(t) + p_{01\to 0}P_{01}(t) + p_{00\to 0}P_{00}(t),$$

$$\frac{dP_m(t)}{dt} = p_{10\to m}P_{10}(t) + p_{00\to m}P_{00}(t), \quad \text{(A7)}$$

where we use the following probabilities of the loci to change their state at time *t*:

$$p_{11\to 01} = 2 \cdot \frac{f_a(1-m)C(t-1)}{C(t)} \cdot \frac{C(t) - f_a(1-m)C(t-1)}{C(t)},$$

$$p_{11\to 00} = \left[\frac{C(t) - f_a(1-m)C(t-1)}{C(t)}\right]^2 \cdot (1 - \frac{1}{N \cdot (1 - C(t-1))}),$$

$$p_{11\to 0} = \left[\frac{C(t) - f_a(1-m)C(t-1)}{C(t)}\right]^2 \cdot \frac{1}{N \cdot (1 - C(t-1))},$$

$$p_{11\to 1} = \left[\frac{f_a(1-m)C(t-1)}{C(t)}\right]^2 \cdot \frac{1}{N \cdot C(t-1)},$$

$$p_{00\to 0} = \left[\frac{1 - C(t) - m}{1 - C(t)}\right]^2 \cdot \frac{1}{N \cdot (1 - C(t-1))},$$

$$p_{00\to m} = 1 - \left[\frac{1 - C(t) - m}{1 - C(t)}\right]^2,$$

$$p_{01\to 00} = \left[\frac{1 - C(t) - m}{1 - C(t)}\right] \cdot \frac{C(t) - f_a(1-m)C(t-1)}{C(t)} \cdot (1 - \frac{1}{N \cdot (1 - C(t-1))}),$$

$$p_{01\to 0} = \left[\frac{1 - C(t) - m}{1 - C(t)}\right] \cdot \frac{C(t) - f_a(1-m)C(t-1)}{C(t)} \cdot \frac{1}{N \cdot (1 - C(t-1))},$$

$$p_{01\to m} = \left[\frac{f_a(1-m)C(t-1)}{C(t)} + \frac{C(t) - f_a(1-m)C(t-1)}{C(t)}\right] \cdot \frac{m}{1 - C(t)}, \quad \text{(A8)}$$

where f_a is the relative fitness of a carrier cell at time *t* - 1, given by:

$$f_a = \frac{1+s}{1 + C(t-1) \cdot s}. \quad \text{(A9)}$$

Here we make the typical assumptions of a coalescence model[3] that generations are discrete and non-overlapping and that the community size (N) is constant throughout time.

After running the coalescence simulation from $t = t_{\text{end}}$ to $t = 0$, we obtain the unconditional probabilities for the two background loci (i) to have coalesced within the simulated time interval ($P_1 + P_0$), or (ii) to be derived from two distinct cells that have either been in the community since $t = 0$, or that have migrated into the patch ($P_{11} + P_{10} + P_{00} + P_{\text{m}}$). In case (i) the two loci are identical and in case (ii) the two loci are different. Only, however, when the two loci descended from 2 distinct cells at $t = 0$ that are carriers (with probability $P_{11}(0)$), we assume that the loci are identical, because the genotype that initially carries the beneficial gene comes into the patch as a clonal group. We can calculate the probability of the two background loci being identical as:

P(2 background loci are identical) = $P_1(0) + P_0(0) + P_{11}(0)$.

The probability of two randomly chosen gene sites to be identical is the Simpson's index of diversity[4]. Here we use the inverse of the Simpson's index to measure diversity, which is the effective number of species in a community (of order 2,[5]). The diversity in the background genome is then given by:

$$D_{bg} = \frac{1}{P(2\ background\ loci\ are\ identical)}. \quad \text{(A10)}$$

Similarly, we can calculate the probability of two focal loci to be identical. Note that two randomly sampled focal loci at $t = t_{\mathrm{end}}$ are identical when they are in two carrier cells (state 11) and different when only one locus is in a carrier cell (state 01). We define the diversity of the focal locus as:

$$D_f = \frac{1}{P(2\ focal\ loci\ are\ identical)}. \quad \text{(A11)}$$

We measure the power of the horizontal sweep using the ratio of the diversity in the background genome over the diversity in the focal locus, and we call this ratio the diversity ratio *DR*, given by:

$$DR = \frac{D_{bg}}{D_f}. \quad \text{(A12)}$$

C) Individual-based model

We develop an individual-based model of our selective patch to confirm our coalescence model prediction and to be able to change the ecological scale of the patch by subdividing the patch into multiple niches. This model allows us to describe an ecologically homogeneous patch where cells live from a single resource, or to model an ecologically heterogeneous patch in which different genotypes are specialized in eating different resources. Alternatively, the ecological subdivision may represent negative frequency-dependent selection of genotypes, for example through phage predation[6]. Our simulated patch contains a fixed number of cells (*N*), where each individual cell is described by a set of three numbers representing the

focal locus (transferable), the genotype of the remaining background genome (non-transferable) and the niche/resource association of the genotype. The background genotype can take any positive integer, which matches the focal locus for cells at the beginning of the simulation and for migrating cells. The focal locus can take the adapted state 1 or alternatively any other positive integer for non-adapted cells.

We start each simulation with a diverse community of cells so that every cell carries a different background genotype (and matching focal locus) with randomly assigned niche association. The initial community then also contains a small clonal group of adapted cells. For our simulations we choose a large community size that is possible to simulate in considerable time ($N = 10^6$) and we choose an initial number of adapted cells so that the frequency process of the adapted type is expected to behave deterministically ($C(0) > 5/(Ns)$),[7], $C(0) = 0.001$). We then update the cell numbers for each following generation according to a discrete time Wright-Fisher process [8]. At each generation the fitness of each cell is determined according to their adaptive state and their competition within the n ecological niches. We simulate the ecological competition similar to the *symsim* model by Friedman et al.[9].

Each cell obtains resources from its assigned niche, but also from the remaining niches. Thus, for a given cell i there is a vector of length n giving the cell's ecological fit to each niche. A cell's competitive strength in its niche is denoted by $z \in [0,1]$, where $z = 1$ means that a cell only competes in its own niche, and $z = 1/n$ means that a cell competes in all niches equally. For example, a cell that is specialized in resources of niche 1 with $z = 0.8$ would compete in each of the n niches (here $n = 5$) according to the vector $\sigma_i = [0.8\ 0.05\ 0.05\ 0.05\ 0.05]$. We then define the competitive weight ω of a cell i in niche j is as the product of its fitness f_i ($f = 1 + s$ for

carriers and f = 1 for non-carrier cells) times its association with niche j, so that ω_{ij} = $f_i\sigma_{i,1}$. For example, the cell specialized in niche 1 has in niche 1 a competitive weight of 0.8 if does not carry the trait, or 0.8(1 + s) if it does carry the trait. Each of the n niches holds a resource share of N/n in each generation, so that a cell obtains resources from niche j proportionally to its relative competitive weight in this niche according to:

$$R_{ij} = \frac{N}{n}\frac{f_i\sigma_{ij}}{\Omega_j}, \quad \text{(A13)}$$

where R_{ij} is the amount or resources obtained by cell i from niche j and Ω_j is the summed competitive weight in niche j given by:

$$\Omega_j = \sum_{i=1}^{N} f_i\sigma_{ij}. \quad \text{(A14)}$$

The total amount of resources obtained by cell i per generation is then given by:

$$R_i = \frac{N}{n}\sum_{j=1}^{n} R_{i,j} = \frac{N}{n}\sum_{j=1}^{n} \frac{f_i\sigma_{ij}}{\sum_{i=1}^{N} f_i\sigma_{ij}}. \quad \text{(A15)}$$

The resources of a cell determine the reproduction of a cell. Because the resource share per niche is scaled to N/n, the mean amount of resources per cell per generation is 1. Therefore, we can use R_i directly as the mean number of offspring of a cell. We update the cell numbers stochastically using a Poisson distribution. Then, cells that lack the selected trait have a chance of acquiring the trait with a probability

$C(t)r$. We use a rate of gene transfer of $r = 10^{-4}$, which is higher than conservative estimates ($10^{-6} \leq r \leq 10^{-5}$,[10]), in order for simulations to run in a reasonable computational time. However, the qualitative effects of niche separation that we observe should generalize to lower rates of genetic transfer. Moreover, recent studies suggest that rates of gene transfer may be orders of magnitude larger than these conservative estimates[11,12]. In simulations with migration, we model this process by replacing existing cells at random with new incoming genotypes (and matching focal locus). Each new genotype is also assigned to an associated niche at random.

Each simulation (implemented in MATLAB) is run for $t_{\text{end}} = 10^5$ time steps, which is equivalent to about 6 years of microbial evolution given a generation time of 30 minutes[13]. We show that the results of our individual-based model match our coalescence model (see Supplementary Fig. 4). We track the background genomes and focal loci of all individuals in the community over time and we also measure the horizontal gene flux by counting the number of gene transfer events. From there we determine the parameters of interest, such as the fraction of carrier cells C, or diversities in different parts of the genome. The diversity of a genome is calculated as:

$$D = {}^{1}\!/_{\sum_{i=1}^{n}(p_i)^2}\,, \quad \text{(A16)}$$

where n is the number of different locus variants present in the community and p_i is their respective proportion. This calculation is analogous to the effective number of species in a community (of order 2,[5]). We measure the power of the horizontal

sweep using the ratio of the diversity in the background genome over the diversity in the focal locus, and we call this ratio the diversity ratio *DR*, given by:

$$DR = \frac{D_{bg}}{D_f}, \quad \text{(A17)}$$

where D_{bg} is the diversity in the background genome and D_f is the diversity in the focal locus. A diversity ratio of 5, for example, means that effectively 5 different background genomes per single focal locus appear in the genomes of the community.

This diversity ratio can also be calculated for compartment *C* and compartment 1-*C* separately. For the compartment of non-carriers (1-*C*) the background genomes match their focal locus, giving a diversity ratio of 1. For the compartment of carriers (*C*), gene transfer allows for an increase of diversity in the background genome against the homogeneous focal locus. Thus, the diversity ratio in *C* is directly linked to the cumulative gene flux. The diversity ratio of the whole community lies between the diversity ratios of compartments *C* and 1-*C*, depending on the size of *C*. It therefore behaves similarly, but not exactly like the cumulative gene flux with respect to a changing selection pressure.

Supplementary References.

1. Mira, A., Ochman, H. & Moran, N. a. Deletional bias and the evolution of bacterial genomes. *Trends Genet.* **17,** 589–596 (2001).

2. Moran, N. a. Microbial minimalism: Genome reduction in bacterial pathogens. *Cell* **108,** 583–586 (2002).

3. Otto, S. & Day, T. *A biologist's guide to mathematical modelling in ecology and evolution*. (2007).

4. Simpson, E. Measurement of Diversity. *Nature* (1949).

5. Hill, M. Diversity and evenness: a unifying notation and its consequences. *Ecology* **54,** 427–432 (1973).

6. Rodriguez-Valera, F. *et al.* Explaining microbial population genomics through phage predation. *Nat. Rev. Microbiol.* **7,** 828–36 (2009).

7. Kaplan, N. L., Hudsont, R. R. & Langle, C. H. The 'Hitchhiking Effect' Revisited. **899,** 887–899 (1989).

8. Sigwart, J. An Introduction to Population Genetics: Theory and Applications.--By Rasmus Nielsen and Montgomery Slatkin. *Syst. Biol.* **63,** 843–844 (2014).

9. Friedman, J., Alm, E. J. & Shapiro, B. J. Sympatric speciation: when is it possible in bacteria? *PLoS One* **8,** e53539 (2013).

10. Wiedenbeck, J. & Cohan, F. M. Origins of bacterial diversity through horizontal genetic transfer and adaptation to new ecological niches. *FEMS Microbiol. Rev.* **35,** 957–76 (2011).

11. Overballe-Petersen, S. *et al.* Bacterial natural transformation by highly fragmented and damaged DNA. *Proc. Natl. Acad. Sci. U. S. A.* **110,** 19860–5 (2013).

12. McCarthy, a. J. *et al.* Extensive horizontal gene transfer during Staphylococcus aureus co-colonization in vivo. *Genome Biol. Evol.* **6,** 2697–2708 (2014).

13. Ulitzur, S. Vibrio parahaemolyticus andVibrio alginolyticus: Short generation-time marine bacteria. *Microb. Ecol.* **1,** 127–35 (1974).
